# Optimal energy management strategy for dual-power coupling tractor based on the adaptive control technology

**DOI:** 10.1371/journal.pone.0292510

**Published:** 2023-11-20

**Authors:** Haishi Dou, Hongqian Wei, Qiang Ai, Youtong Zhang

**Affiliations:** 1 School of Mechanical Engineering, Beijing Institute of Technology, Beijing, China; 2 Low Emission Vehicle Research Beijing Key Laboratory, Beijing Institute of Technology, Beijing, China; Vellore Institute of Technology, INDIA

## Abstract

Hybrid tractors (HT) are regarded as the efficient agricultural machine due to their energy conservation performance and faster torque response to deal with load fluctuations. However, the strategy to allocate the battery and fuel energy for demand power should be discussed. In this paper, an on-line management strategy of the HT is proposed to optimize the energy consumption of engine and motor and to reduce torque ripple for power units. A new architecture for replacing power shift and continuously variable transmission technology is proposed. Then, the modified equivalent consumption minimization strategy (ECMS) is used to optimize the torque distribution in which the equivalent factor is further calculated for the real-time process. Besides, the modification of ECMS in variable working conditions can effectively analyse the torque distribution between the motor and engine. The numerical test is implemented that the effectiveness of the proposed energy strategy is validated in plowing conditions. The consequences indicated that the proposed power distribution strategy can adaptively allocate the torque demand according to the fluctuation load. Comparing with the traditional rule-based strategy, the proposed strategy can reduce 6.2% of the energy, and decrease torque ripple with the proposed tractor architecture.

## Introduction

Hybrid electric tractors play an important role in agricultural production which the energy resources are come from fossil and electricity. Fossil energy is available in power generation, and that is effective in environmentally friendly [1–3]. So, hybrid electric tractor has produced bright prospects with new power system, which they can operate efficiently in agricultural production [3, 4]. Besides, suitable energy management strategy (EMS) could allow motor and engine to operate at high efficiency levels, increasing the tractor efficiency [5, 6]. The architecture of the tractor should satisfy the various operation mode, such as rotary tillage working conditions with the power take-off (PTO) is conducted. The orchard tractor with a parallel hybrid electric powertrain, which including three kinds of different duty are proposed [7, 8]. The hybrid system tractors are proposed, which is driven by hybrid power system. The hybrid architectures were configured with the battery and fuel. Most of those powertrains’ architectures consisted of the hybrid power system. On the other hand, the traditional tractors couple the PTO and drive wheel power by the gearbox transmission, which can make the rotation speed of the PTO match with the forward velocity of the electric tractor [9].

As for EMS used on tractors, the EMS of hybrid electric tractor can be divided into two categories, that was, rule-based and optimization-based. The effective energy management strategy of dual-power coupling tractor (DPCT) could enlarge the mileage with carried energy which ensured dynamic of tractors. As for optimization-based, the particle swarm optimization algorithm strategy was used in the optimized process. Then the driving situations indicated energy cost of hybrid electric vehicles could be reduced by 9.88% in certain driving cycles [10]. A similar optimization method could be used in hybrid electric vehicles [11]. To improve the energy efficiency of hybrid powertrains, the short-term load prediction based on Bayesian inference, and a cycle detection based on correlation were offered and analyzed [12]. The nonlinear model predictive control that combines an accurate control-oriented model and a dynamic process coordination control algorithm was researched [13]. And the control laws were solved as a multi-parameter quadratic programming optimization problem. The torque distribution strategy were obtained by offline solving the multi-parameter quadratic programming. A combined short-term forecasting model for driving conditions was built that included stochastic forecasting and machine learning [14]. Based on the prediction of demand power, an effective convergent offline learning controller was created with the vehicle energy management feature. Besides, a vehicle speed prediction model based on the Markov algorithm was created to anticipate the speed of the next 5s, which was proposed to optimize the power allocation [15, 16]. In general, these methods attempted to forecast the power needed of machines and allocate fuel and electric energy appropriately. As for rule-based optimization algorithm, the rule-based method was employed to define the mode switching border. The charge-depleting mode (CD) of the rule-based energy strategy was then replaced with an instantaneous optimization method to discover the real-time optimal solution [17]. As for agriculture machines, the tractor operated approximately at a known speed except with load fluctuations, so the charge-sustaining of rule-based could be trial in a specific driving cycle [18]. The architecture tractors conducted in low speed and specific working times in whole day, so state of charge (SOC) could be scheme in multiple driving cycles by rule-based [19, 20]. The plug-in hybrid electric vehicle has achieved range-extended by 9% through charge-sustaining and charge consumption strategy [21]. In summary, these rule-based optimization methods had the merits of tractors efficient, but the shortcomings were the online performances. And the rule–based has the demerits of facing with variable parameter operating conditions. So this paper proposed the modified ECMS. And this method was promising to satisfy the power demand and efficiency of the tractors.

The prior references examined the energy efficiency of hybrid powertrains, the two points should be considered. Firstly, the proposed strategy should consider the load fluctuation of the actual tractor’s operating conditions. Consequently, the varying impact load would affect the dynamics and they may result in poor performance of EMS. Secondly, the architecture of the traditional powertrain coupling the PTO and drive system, that is an obstacle to further improving the operation quality. Therefore, the EMS of PTO and the rear driven system output independently should be further researched.

Comparing with other EMS, the proposed strategy could integration the online torque distribution more easier. And the strategy is adaptive both tractors rotary tillage and plowing operation mode. Besides, the other EMS is designed for similar configurations, but the proposed strategy is researched for new configuration tractor.

## The dynamics system and power architecture of dual-power coupling tractor

**Fig 1:**
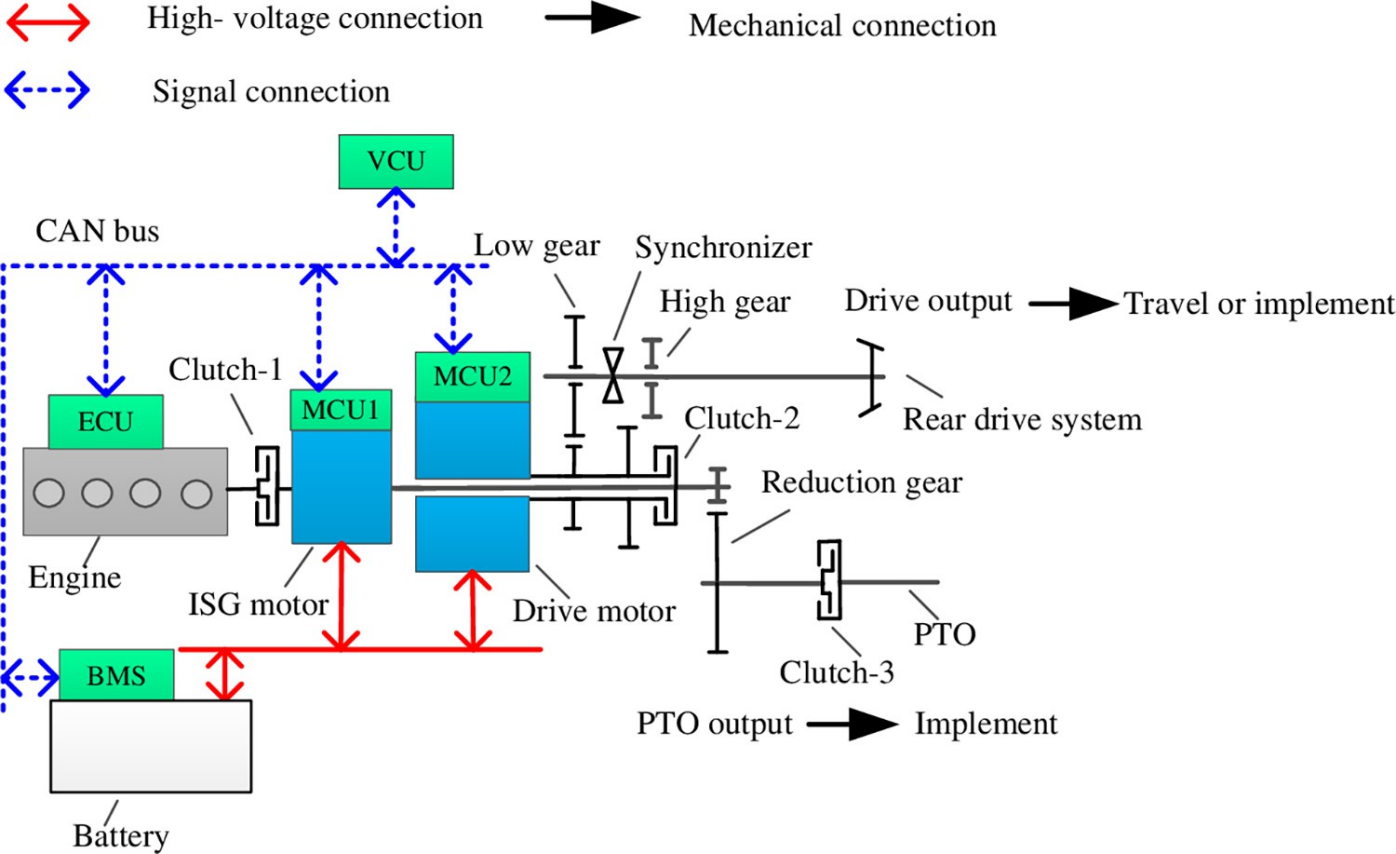
The proposed architecture of dual-power coupling tractor.

This paper proposed the dual-power coupling tractor (DPCT) that is a kind of hybrid electric tractors. As a new architecture of the tractor with multiple driving modes, such as engine working alone, engine and drive motor working coupling, integrated starter and generator (ISG) motor, and drive motor working coupling, all of those modes could be conducted efficiently by an excelled EMS with the dynamic working condition. The architecture is about a power coupling or decoupling tractor for PTO and rear driving system, which the architecture diagram is shown in Fig 1. The dual-power coupling tractor consists of the engine and motor. What is more, the obvious difference between road vehicles and DPCT are the PTO system. So EMS should be researched for both power units and tractor implements drive systems.

## Dynamic vehicle system

### Architecture and operation mode of the dual-power coupling tractor

The architecture of DPCT is shown in Fig 1, the power coupling or decoupling between the ISG motor and drive motor is depending on clutch-2. Especially, the output shaft of the drive motor is hollow. The power flow of the ISG motor is connected to the rear drive system and PTO. Meanwhile, the output shaft power of the drive motor could be selected between low gear and high gear that is chosen on the motors’ high-efficiency range and then transmitted to the front and rear drive system. And the output speed of ISG motor is decelerated by the reduction gear and then transferred to PTO. Besides, the engine power is connected with ISG motor by clutch-1. To improve the fuel efficiency of the engine, the ISG motor plays a role in a power regulating unit between the electric drive and the power generator. The parameters of power units of tractors are shown in Table 1. As for tractor control system, the motor control unit (MCU), engine control unit (ECU) and battery management system (BMS) are connect with controller area network (CAN) bus, which is exchange data with vehicle control unit (VCU). The traditional architecture of tractor is equipped only one high-engine and the complicated reduction gearbox, and transfer power to PTO and rear drive system. Both the demand power of implement and travel are from engine, when dealing with different power demands, the overall efficiency of the tractor can be further improved.

**Table 1:** Parameters of tractor power unit.

	Rated power (kW)	Rated speed (rpm)	Rated torque (Nm)
Drive motor	90	3000	290
ISG motor	65	2200	280
Engine	140	2100	630

#### Longitudinal dynamics

In this powertrain system, the working load includes acceleration resistance *F*_*A*_, the rolling resistance *F*_*r*_. Besides, including the gradient resistance *F*_*g*_, in the condition of the tractor implement load is not participating in the power system. The longitudinal dynamics equations of DPCT is shown as follows [22].

$$P_{dem}=(F_{A}+F_{r}+F_{g})V_{x}$$ (1)

Where *P*_*dem*_ indicates the total demand power of working load. And the load resistances are present as.

$$F_{g}=Mg\times \sin\theta$$ (2)

$$F_{r}=Mg\times \cos\theta \times r_{r}$$ (3)

$$F_{A}=M\frac{dv}{dt}$$ (4)

$$P_{dem}=P_{e}+P_{i}+P_{d}$$ (5)

Where *θ* is the slope of the unstructured roads. *r*_*r*_ is the rolling resistance.

#### Operation mode analysis and description

**Fig 2:**
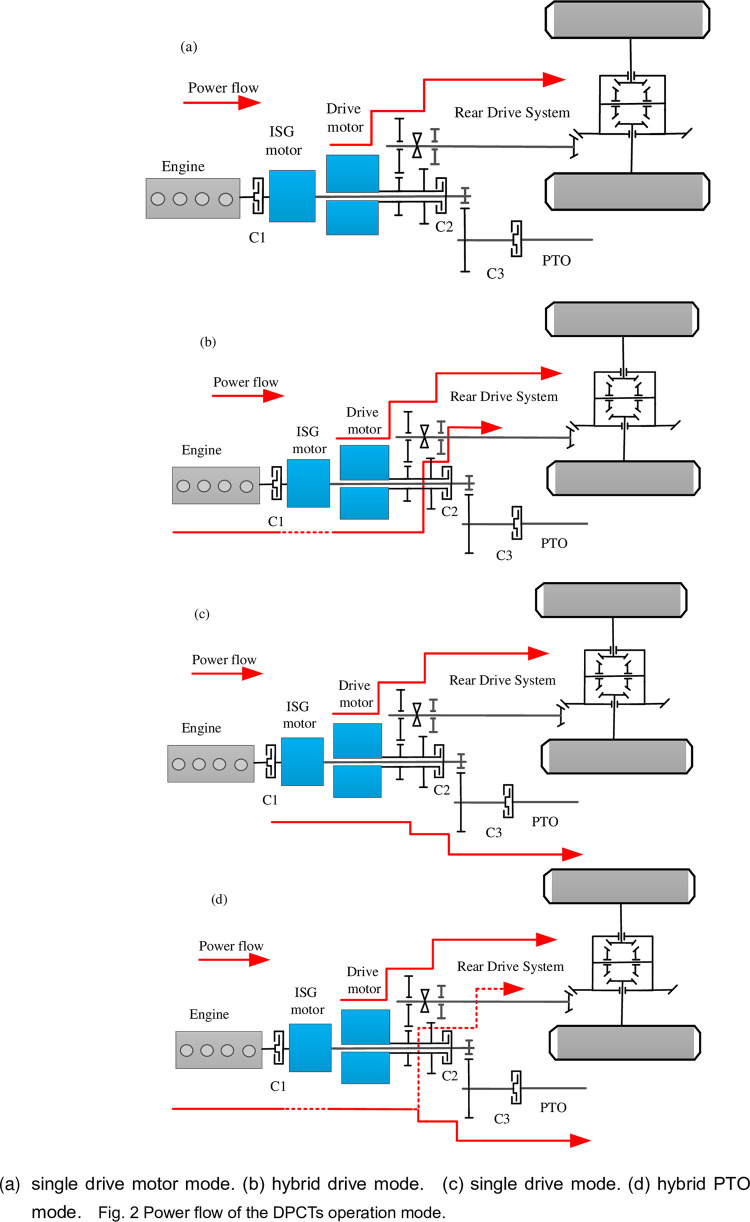
Power flow of the DPCTs operation mode.

To reasonably analyzing the process of power flow transmission, the operation mode could be classified into two kinds of terminal drive such as to rear drive system and to PTO system. Especially, to rear drive system could consist of single drive motor mode (SM1). To ensure the engine works at a high-efficiency range, the engine and ISG motor are used to range extender mode incorporated in the rear drive system. Additionally, the ISG motor is not in power generation mode, and the engine is engaged in the rear drive system which is composed of hybrid drive mode (HDM). Besides, when the tractor works in rotary tillage conditions, the drive motor plays the role of driving and the ISG motor works as the output power for PTO, which is the mode of single drive mode (SM2). What is more, the engine will engage with the increase of demand power of PTO that is in hybrid PTO mode (HPM). Furthermore, to more thoroughly explain each mode, the clutch and power unit states are shown in Table 2, and the power flows in plowing or rotary tillage mode are illustrated in Fig 2.

**Table 2:** The operation mode of the DPCTs.

	Modes	Engine	ISG Motor	Drive motor	C1	C2	C3
(a)	SM1	Off	Off	drive	Off	Off	Off
(b)	HDM	On	drive/power generation	drive	On	On	Off
(c)	SM2	Off	drive	drive	Off	Off	On
(d)	HPM	On	drive/power generation	drive	On	On/Off	On

According to the above analysis, the dynamic equations of transmission in plowing mode could be expressed in:

$$[\begin{array}{c} w_{e}\\ w_{i}\\ w_{d} \end{array}]=[\begin{array}{c} i_{0}i_{1}\\ i_{0}i_{1}\\ i_{0}i_{1} \end{array}]w_{w}$$ (6)

$$\left[\begin{array}{ccc} 0 & 0 & J_{d}\\ J_{e} & -J_{i} & J_{d} \end{array}][\begin{array}{c} {\dot{w}}_{e}\\ {\dot{w}}_{i}\\ {\dot{w}}_{d} \end{array}]=[\begin{array}{cccc} 0 & 0 & 1 & -\frac{1}{i_{0}i_{1}}\\ 1 & -1 & 1 & -\frac{1}{i_{0}i_{1}} \end{array}][\begin{array}{c} T_{e}\\ T_{i}\\ T_{d}\\ T_{out} \end{array}\right]$$ (7)

In this process, the ISG motor is worked as electric generate appropriately, based on SOC and dynamic load that is affected by the parameters of soil and load. Meanwhile, when the DPCT is used in rotary tillage mode, the dynamic equations of transmission could be described as follows:

$$\left[\begin{array}{c} w_{e}\\ w_{i}\\ w_{d} \end{array}]=[\begin{array}{ccc} i_{0}i_{1} & 0 & 0\\ 0 & i_{3} & 0\\ 0 & 0 & i_{0}i_{1} \end{array}][\begin{array}{c} w_{w}\\ w_{\mathrm{pto}}\\ w_{\mathrm{pto}} \end{array}\right]$$ (8)

$$\left[\begin{array}{ccc} 0 & J_{i} & J_{d}\\ J_{e} & -J_{i} & J_{d} \end{array}][\begin{array}{c} {\dot{w}}_{e}\\ {\dot{w}}_{i}\\ {\dot{w}}_{d} \end{array}]=[\begin{array}{ccccc} 0 & 1 & 1 & -\frac{1}{i_{0}i_{1}} & -\frac{1}{i_{3}}\\ 1 & -1 & 1 & -\frac{1}{i_{0}i_{1}} & -\frac{1}{i_{3}} \end{array}][\begin{array}{c} T_{e}\\ T_{i}\\ T_{d}\\ T_{out}\\ T_{pto} \end{array}\right]$$ (9)

#### Tractor dynamics load

In the condition of the tractor in load, the power units of DPCT are used to overcome rolling resistance and load resistance from tractor implements in plowing conditions. According to the drive and resistance moment balance of dynamic, the mathematical formulation of the load resistance could be expressed in [23]:

$$F_{t}=z\cdot b_{0}\cdot h_{0}\cdot k_{0}$$ (10)

$$F_{T}=F_{t}+0.5\cdot F_{t}\gamma_{1}\sin(\tau_{1}t)$$ (11)

$$F_{f}=fMg\cos\theta$$ (12)

$$P_{T}=\frac{(F_{t}+F_{f})\cdot v_{x}}{3600}$$ (13)

Where the *z* and *b*_0_ indicate the number of plowshares and plowshare width, respectively. *h*_0_ is the tillage depth which varies with undulation of the ground. And the *k*_0_ is the soil proportion resistance that is changed with soil parameters. *f* is the rolling resistance coefficient in the land. The dynamic load is affected by tractor implements resistance and the rolling resistance. Considering the real soil environment, the tractors’ resistance is variable along with operation time and location. So, the actual resistance during operation is expressed as *F*_*T*_, where the *γ*_1_ is the uneven rate of traction resistance, *τ*_1_ is the frequency of resistance change of agricultural machinery, and *t* is the tractor operation time.

In the condition of rotary tillage of tractor, the power between PTO and traveling drive system are departure. What is more, the PTO is working in a constant speed range between 700~1200rpm, and the implement load resistance of rotary tillage based on the soil-specific resistance method is calculated, which is shown below [24].

$$F_{r}=0.1(K_{g}K_{1}K_{2}K_{3}K_{4})hB$$ (14)

In considering actual working conditions, the implement load resistance is rewritten as follows [25].

$$F_{R}=F_{r}+0.5F_{r}\gamma_{2}\sin(\tau_{2}\cdot t)$$ (15)

Where the *τ*_2_ is the frequency of resistance change of agricultural machinery, *γ*_2_ is the uneven rate of traction resistance in rotary tillage and transfer conditions [26].

### Electric drive system

**Fig 3:**
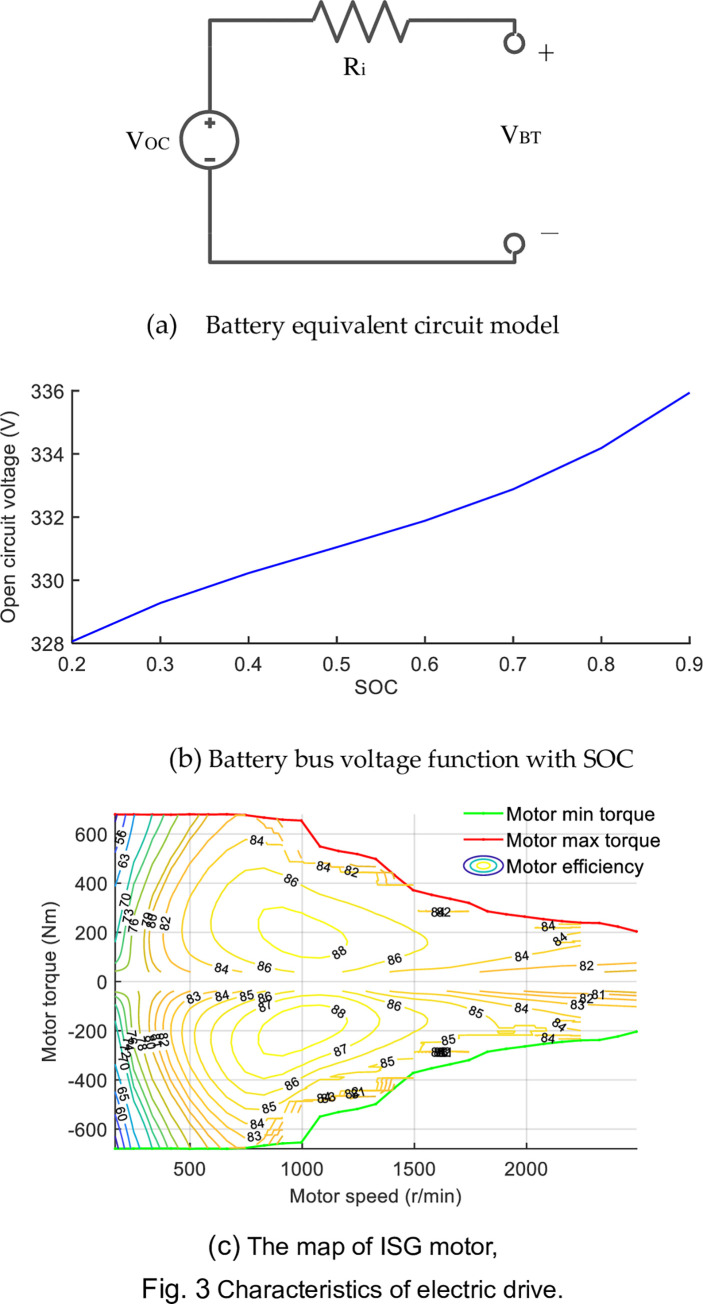
Characteristics of electric drive. (a) Battery equivalent circuit model. (b) Battery bus voltage function with SOC. (c) The map of ISG motor.

The Lithium-ion battery packages provides electric energy for the drive motor, to reduce the number of battery packages, the additional power is supplied by the engine, which ISG motor is worked in electric generator mode. So the SOC can maintain the range from 30% to 90% mostly to sustain the health of the battery. The model of the battery pack is composed of the voltage source and the internal resistance [27]. The functional relationship between bus voltage V_oc_ and SOC is tested in offline experimental, which is shown in Fig 3(A) and 3(B). The variations between battery SOC’s and bus current *I*_*BT*_ are expressed as follow [28].

$$SOC(t)=\frac{1}{Q_{0}}\int_{t_{0}-1}^{t_{0}}I_{BT}(SOC(t-1),sign(P_{bat}))dt+SOC(t-1)$$ (16)

Where the *Q*_0_ is maximum battery capacity and the *SOC*(*t*−1) is the battery level at the previous moment. The following equations depict the relationship between the power battery’s bus voltage, internal resistance, output power, and bus current [29].


$$I_{BT}\left(SOC,sign(P_{{}_{bat}})\right)=\frac{V_{oc}(SOC)-\sqrt{V_{oc}^{2}(SOC)-4\times R_{\mathrm{int}}(SOC,sign(I_{BT}))\times P_{{}_{bat}}}}{2\times R_{\mathrm{int}}(SOC,I_{BT})}$$ (17)

$$P_{{}_{bat}}=V_{BT}\times I_{BT}=V_{oc}\times I_{BT}-R_{\mathrm{int}}\times I_{BT}^{2}$$ (18)

$$V_{BT}(SOC,I_{BT})=V_{oc}(SOC)-R_{\mathrm{int}}(SOC,I_{BT})\times I_{BT}$$ (19)

Permanent magnet synchronous motors (PMSMs) are employed in both drive and generator mode in ISG motors. The driving motor is also a kind of PMSM. Motor characteristics are investigated on an experimental platform. Similarly, the motor map can be used to compute total motor power based on rotation speed, motor torque, and electric efficiency.

$$P_{m}=\frac{T_{e}\times n}{9550}\times \eta^{p}(T_{e},n)$$ (20)

Where *p* is the indicator of tractor-driving mode. When *p* = −1, the motor is in generation mode; otherwise *p* = 1, the motor is in driving mode. The ISG motor efficiency can be test and record, and the efficiency map is obtained in Fig 3(C).

## Energy management strategy

The main contribution of an energy management strategy is to allocate fuel and electric power appropriately to minimize equivalent fuel consumption [30, 31]. Besides, equivalent fuel consumption costs are established in the cost-optimal control problem in this paper. What is more, the equivalent fuel consumption cost is reflecting the allocated laws of DPCT in the dynamic load that is affected by torque ripple. The electric power cost is embodied by SOC, since the SOC is crucial in the range of the drive cycle and maintains battery service life. Besides, the shock of DPCT is affected by differences in torque between the engine and motors when the power coupling or decoupling are in a different working mode. Thus those influence factors are redesigned in EMS.

### Adaptive equivalent consumption minimization strategy

**Fig 4:**
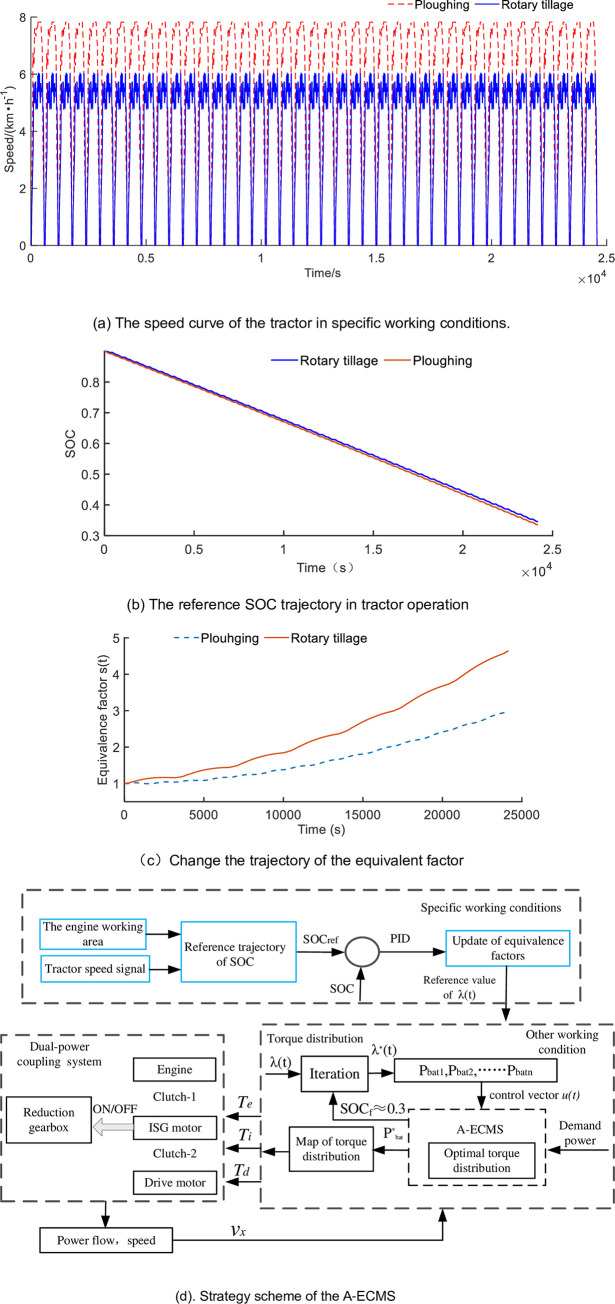
The optimized process in the tractors’ operation. (a) The speed curve of the tractor in specific working conditions. (b) The reference SOC trajectory in tractor operation. (c) Change the trajectory of the equivalent factor. (d). Strategy scheme of the A-ECMS.

The A-ECMS is a detailed modification of the equivalent consumption minimization strategy (ECMS), which the ECMS is described in reference [32]. In this paper, the power distribution is going on particular working conditions, firstly, the tractor speed is going as Fig 4(A), which is consisted of multiple driving cycles, and the ECMS is described as follows.


$$J={\dot{m}}_{eqv}(t,u)={\dot{m}}_{fuel}(t,u)+{\dot{m}}_{bat}(t,u)$$ (21)

$${\dot{m}}_{fuel}(t)=\frac{1000P_{e}(t)}{\eta_{e}Q_{lhv}}$$ (22)

$${\dot{m}}_{bat}(t,u)=s(t)\frac{P_{bat}(t,u)}{Q_{lhv}}$$ (23)

The ${\dot{m}}_{eqv}(t,u)$ is the equivalent fuel consumption rate of the engine and battery, ${\dot{m}}_{fuel}(t,u)$ is the fuel consumption rate of the engine, the ${\dot{m}}_{bat}(t,u)$ is the conversion of battery power consumption into fuel consumption rate, as shown in Eq (24).

$${\dot{m}}_{bat}(t,u)=s(t)\frac{P_{bat}(t)}{Q_{lhv}}=s(t)\frac{V_{BT}Q_{0}}{Q_{lhv}}\frac{I_{BT}}{Q_{0}}$$ (24)

The *P*_*bat*_(*t*,*u*) is the discharge power of the battery, and the *Q*_*lhv*_ is the low calorific value of the fuel. *s*(*t*) is the equivalent factor, in the conversion process:

$$\lambda (t)=-s(t)\frac{V_{BT}Q_{0}}{Q_{lhv}}$$ (25)

$${\dot{m}}_{bat}(t,u)=-\lambda (t)\frac{I_{BT}}{Q_{0}}=\lambda (t)S\dot{O}C$$ (26)

And considering the load fluctuations in actual working conditions, the objective function is rewritten as:

$$J={\dot{m}}_{fuel}(t,u)+\lambda (t)S\dot{O}C+\frac{T_{e\_\max}(t)-T_{e\_\min}(t)}{T_{e\_\max}(t)+T_{e\_\min}(t)}$$ (27)

The last term in above equation is used to optimize the range of engine output torque, which reduces engine torque fluctuations. *λ*(*t*) is the equivalent factor after transformation. *T*_*e_max*_ and *T*_*e_min*_ is the maximum and minimum torque output of the engine, respectively. So the high-frequency load fluctuations could be overcame by motors.

The solving process is carried out. Firstly, the speed and load resistance of the tractor is known in advance under specific operating conditions, so the engine is working in a range with thermal efficiency greater than 28%, and the ISG motor and drive motor are adjusted with load fluctuation. The curve of SOC is designed in specific operating conditions, and the equivalent factor is determined by iteration. Second, as for variable operation conditions, such as variable speed and other types of tools of the tractor, the equivalent factor is tracking the specific operation conditions then, the objective function is solved by the Hamiltonian function.

When the ISG motor is working in power generation mode, the variation of SOC and power generation is defined as follows.

$$W_{bat}=\int P_{bat}dt$$ (28)

$$\Delta SOC=\frac{W_{bat}\eta_{ch}}{3.6\cdot V_{OC}Q_{\mathrm{bat}}}$$ (29)

Where the *Q*_bat_ is battery capacity. The specific working condition is known in advance. In the transmission between the power unit and load resistance, the power system existed the highest efficiency point in tractor working. The control vector *u* is defined as follows.

$$u=\arg\;\min\{\int_{t_{0}}^{t_{f}}(P_{e}(t)+P_{bat}(t))dt|v_{x},P_{dem}\}$$ (30)

$$P_{bat}(t)=\frac{P_{d}(t)}{\eta_{d}}\pm P_{i}(t)\eta_{{}_{{}_{i}}}^{p}$$ (31)

When *p* = −1, the ISG motor is in generation mode; otherwise *p* = 1, the ISG motor is in driving mode. *P*_*i*_(*t*) and *η*_*i*_ are the power of ISG motor and efficiency respectively. The working point of the engine is determined as known, and the ISG motor and drive motor is determined by search. During this process, the power of the battery is defined as a control vector. Then, picking out the optimal vector in the candidate vector of battery power.


$$P_{bat}^{*}=\arg\;\min\{\int_{t_{0}}^{t_{f}}(P_{e}(t)+\frac{P_{d}(t)}{\eta_{d}}\pm P_{i}(t)\eta_{{}_{{}_{i}}}^{p})dt|v_{x},P_{dem}\}$$ (32)

### Reference trajectory design in specific operating conditions

The specific operating conditions proceed as Fig 4(A). So the speed of the tractor is known in advance, and the distribution of electric and fuel energy could be determined by searching and iteration. Firstly, the engine operates in a range with a thermal efficiency greater than 28%, then distributes the output power of the battery, and determined the electric power by the Eq (32). Considering the number of cycles of the battery, the SOC of the battery is restricted between 0.3 and 0.9. So the curve of SOC is designed in specific operation conditions, as shown in Fig 4(B), which is solved by iteration. Furthermore, the trajectory of the equivalent factor is defined during the day’s homework process. The working hours of the tractor are defined as 6.5 hours a day. The equivalent factor is determined by Eq (25). In the full electric power and fuel in a day’s operation of the tractor, the equivalent factor is calculated and shown in Fig 4(C) in specific working conditions. Comparing the equivalent factor was set as a constant, that the variable equivalent factor could balance the battery consumption with the decline of SOC and protect the cycling life of the battery. The scheme of the A-ECMS strategy is shown in Fig 4(D).

As for other operation working conditions, such as variable tractor speed and other kinds of working implement, the equivalent factor could be trailed. The nonlinear PID algorithm is used in trailing equivalent factor trajectories. The specific process is defined as follows.

$$\lambda (t)=\{\begin{array}{ccc} K_{p}(SOC_{ref}-SOC)+K_{i}\int \left(SOC_{ref}-SOC\right)dt & \mathrm{if} & \lambda (t)>0\\ \lambda (t-1) & \mathrm{if} & \lambda (t)<0 \end{array}$$ (33)

Where *K*_*P*_, *K*_*i*_ is the proportional and integral coefficients, respectively. The value of *K*_*P*_, *K*_*i*_ are affected by properties of first-order systems, which is defined by bench test. And there are defined by characteristics of the system. *SOC*_*ref*_ is the reference trajectory of SOC in specific working conditions. The SOC in equations is replaced and update in battery management system. The *λ*(*t*) is the equivalent factor that could be adaptively adjusted in other working conditions.

## Bench testing and discussions

**Fig 5:**
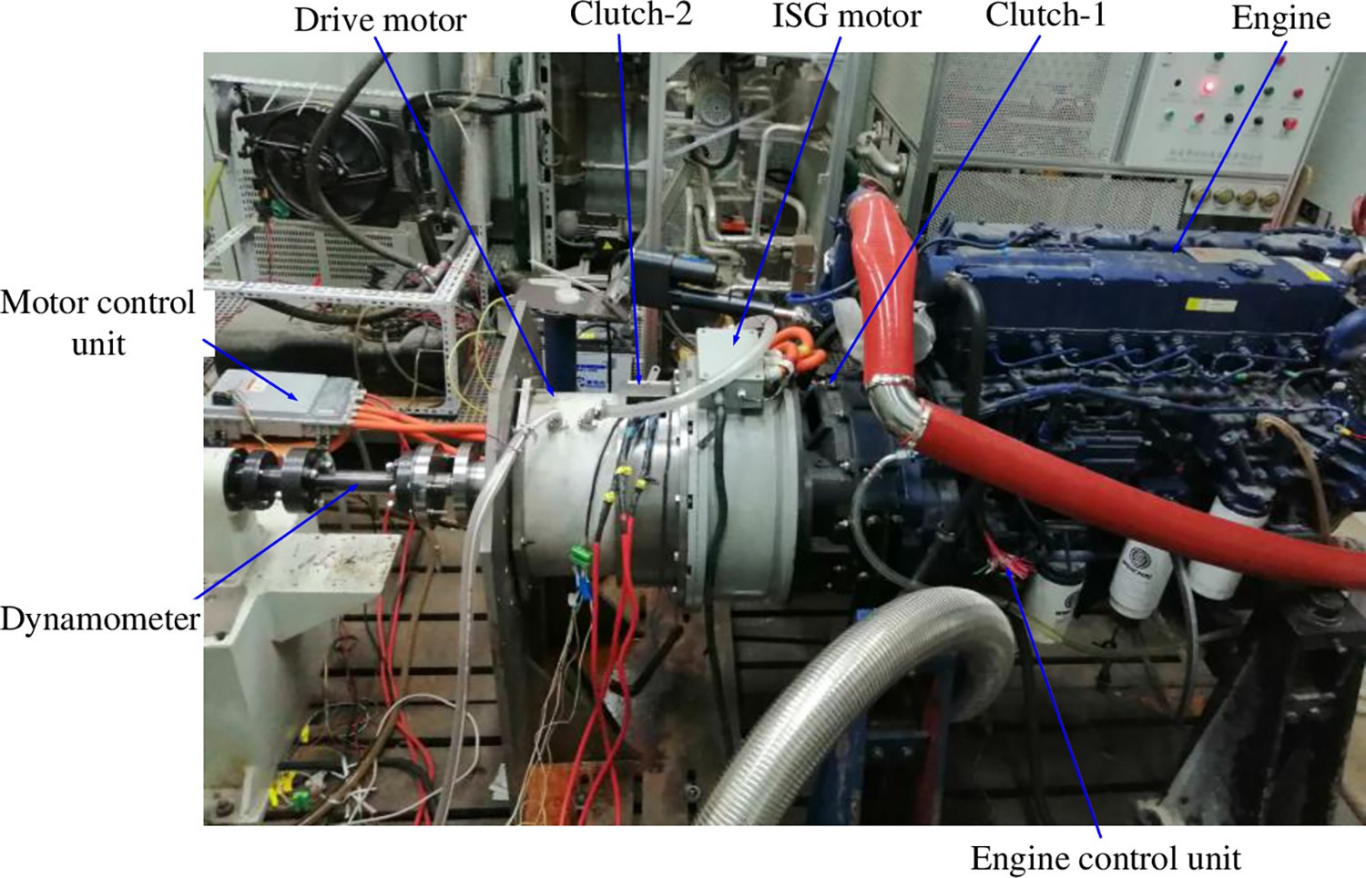
The test platform of DPCT power system.

The clutch-2 and drive motor is designed and manufactured which is used to demonstrate the effectiveness of the proposed energy management strategy for DPCTs. And the power system of the proposed architecture as Fig 1 is assembled. The test platform is shown in Fig 5. Specifically, the test platform includes a dynamometer, a vehicle control unit (VCU), a motor control unit (MCU), an engine control unit (ECU), and the controller area network (CAN) communication devices. The primary states of power units can be recorded. The proposed energy management approach can adaptively allocate output power between the engine and motor using the integrated strategy by reading up table written to VCU. Furthermore, the computer can store the state variables and parameters [33]. To demonstrate the effectiveness of the proposed technique, the typical rule-based optimization algorithm is employed for comparison. The process of rule-based algorithm is working as charge-sustaining state. The parameters of discussed DPCT and the corresponding tractor agricultural tools are listed in Table 3.

**Table 3:** Parameters of DPCT and tractor agricultural tools.

Description	Values	Description	Values
Total tractor mass *M*	13100 kg	Coefficient of rolling resistance *f*	0.06
Battery capacity *Q*_0_	95 kW.h	Plow width *b*_0_	25 cm
Open circuit voltage *V*_*BT*_	340 V	Resistance coefficient of plowing *k*_0_	3 ~7.5 N/cm^2^
Plowing speed *v*_*p*_	5~10km/h	Plowing depth *h*_0_	20~40 cm
Rotary tillage speed *v*_*r*_	2~8 km/h	Resistance coefficient of rotary tillage *K*_*g*_	11~13 N/cm^2^
Number of plowshares z	8	Rotary tillage width *B*	2.1~3 m

### The map of torque optimization in specific working conditions

**Fig 6:**
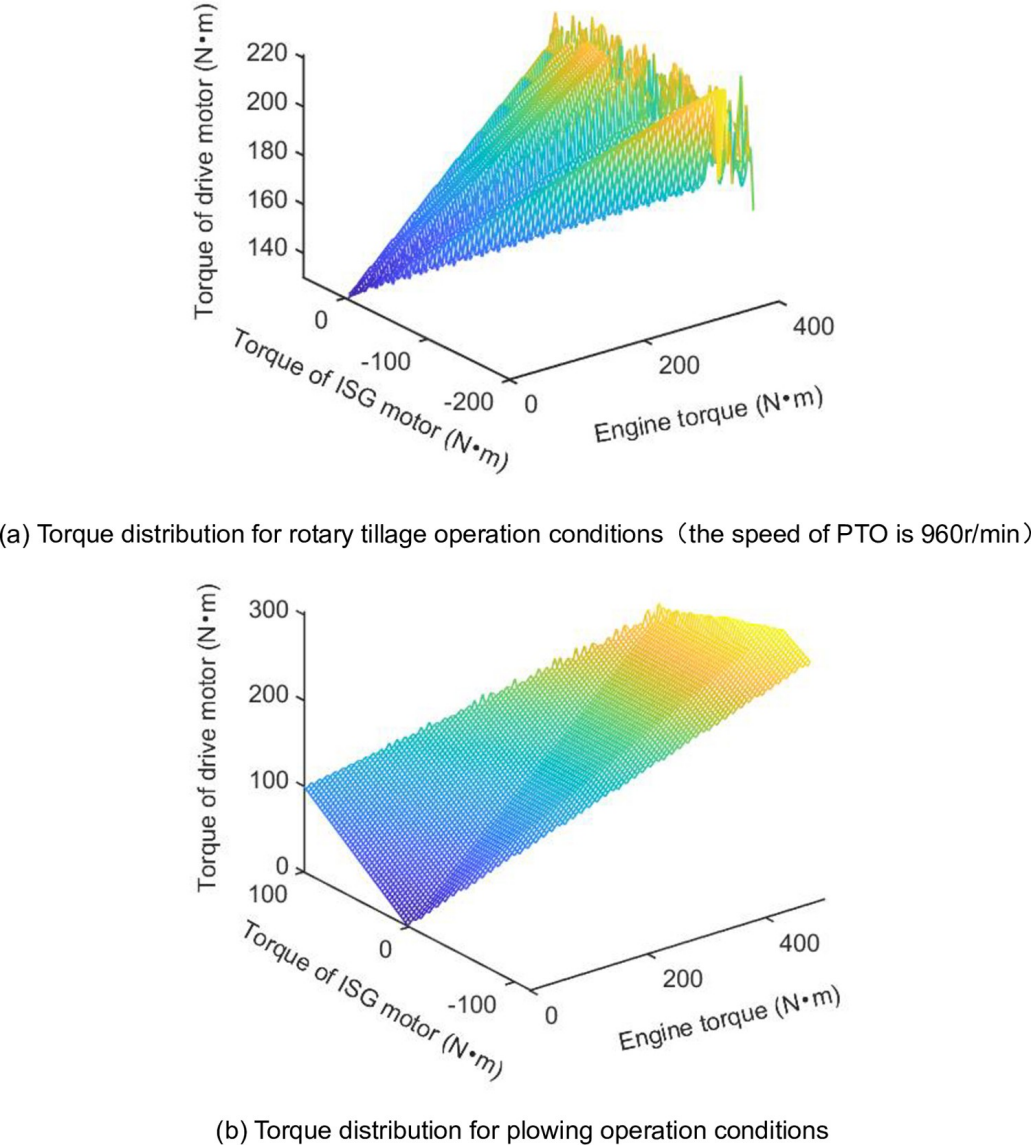
The map of optimal torque distribution for power units. (a) Torque distribution for rotary tillage operation conditions (the speed of PTO is 960r/min). (b) Torque distribution for plowing operation conditions.

The characteristics of the energy management strategy are expressed in Fig 4(D). In particular, the tractor carries battery fewer, the function of the engine not only outputs torque to PTO but also provides power for the ISG motor to generate electricity, and to maintain the tractor operators 6.5 hours a day. The ISG motor not only plays a role in generators to maintain the SOC value around 0.3 at the end of work but also serve as a driving motor. In the specific driving conditions, the battery reference SOC curve and equivalence factor are known, so the optimal torque distribution map in plowing and rotary tillage operation is shown in Fig 6. Fig 6(A) and 6(B) depict the power units distribution between engine and motor with the proposed EMS. The details in Fig 6(A) show the engine operates at around 410Nm and 1500r/min. The engine is working in the efficient stable range and the ISG motor dealing with load fluctuations during the PTO working process. But the drive motor outputs independently to drive the tractor to move. On the other hand, the power flow to tractors’ operating tools is driven by the engine, and the speed of the PTO shaft is around 960r/min.

Fig 6(B) illustrates the torque distribution of the engine and motor in plowing operation mode. The ISG motor also operates in power generation mode. The torque distribution map is optimized by iteration in known conditions. In the working of plowing conditions, the power flow of plowing proceeds as Fig 2(B). The engine operates point at around 460Nm and 1800r/min, and the drive motor to dealing with load fluctuations.

### Case 1: Variable operating condition analysis based on tractor plowing operation project

In this section, the DPCT is controlled by A-ECMS. Accordingly, the variable operating conditions are particularly analyzed. For the variable tractor speed and other types of tractor tools, the adaptive torque distribution strategy is researched in tractors operation mode.

**Fig 7:**
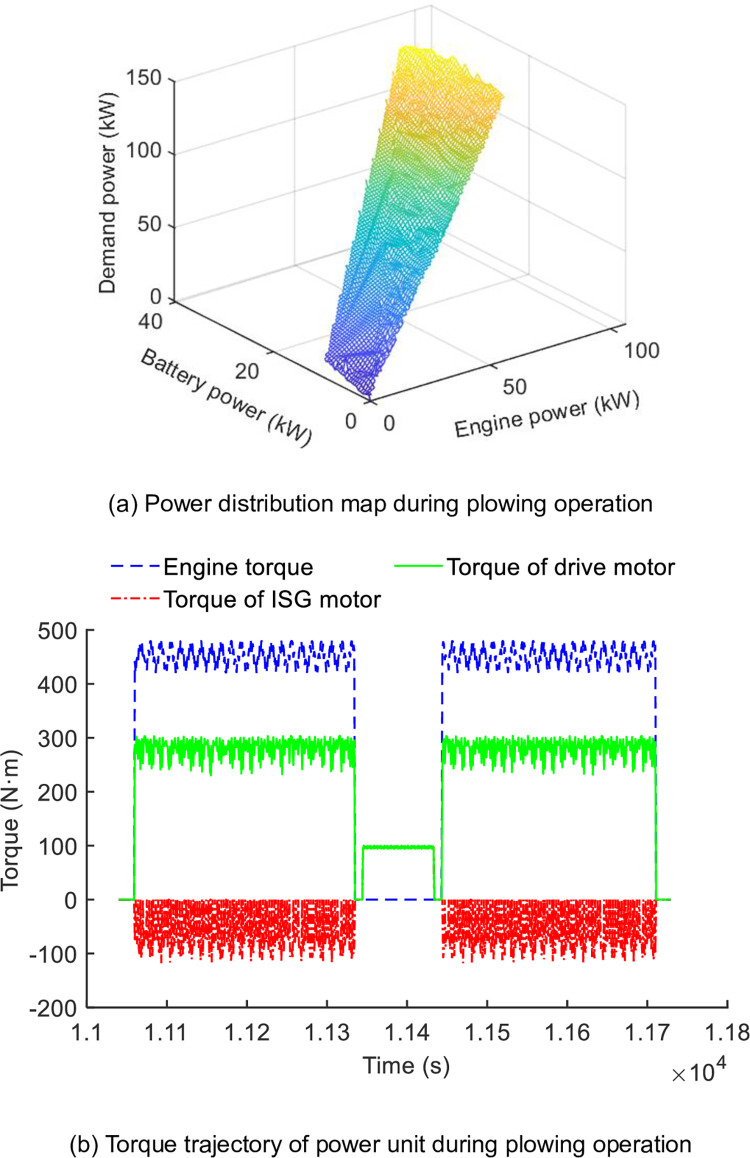
Power distribution in plowing and rotary tillage working conditions. (a) Power distribution map during plowing operation. (b) Torque trajectory of power unit during plowing operation.

The torque distribution of the engine and motors in variable operating conditions are depicted in Fig 7(A) and 7(B). The proposed EMS is inclined to allocate the demand power corresponding with electric power and engine power. In detail, in facing an increase in load from the implement, the equivalence factor *λ*(*t*) is referenced by specific working conditions, then discretizing the battery output power, and determining the battery output power through the minimum value of the Hamiltonian function. If the final value of SOC is around 0.3 in the iteration process, the equivalence factor *λ*(*t*) do not needed to adjusted, otherwise, changing the value of the equivalent factor *λ*(*t*) in steps of 0.2. The power distribution relationship between the engine and battery determined based on the minimum value of the Hamilton function is shown in Fig 7(A).

As shown in Fig 7(A), the map has optimized the output power between electricity and fuel along with the demand power increase. In detail, in the one type of torque distribution shown in Fig 7(B), the engine is working at an efficient range, the ISG motor is in generator mode to supply electric energy, and the torque of the drive motor fluctuates with a load. The torque of power units is balanced with the tractor load. In the period of 1.135*104s-1.145*104s, the tractor is turning around, and the drive motor and ISG motor provide power to overcome load resistance.

### Case 2: Comparison with rule-based energy management strategy

**Fig 8:**
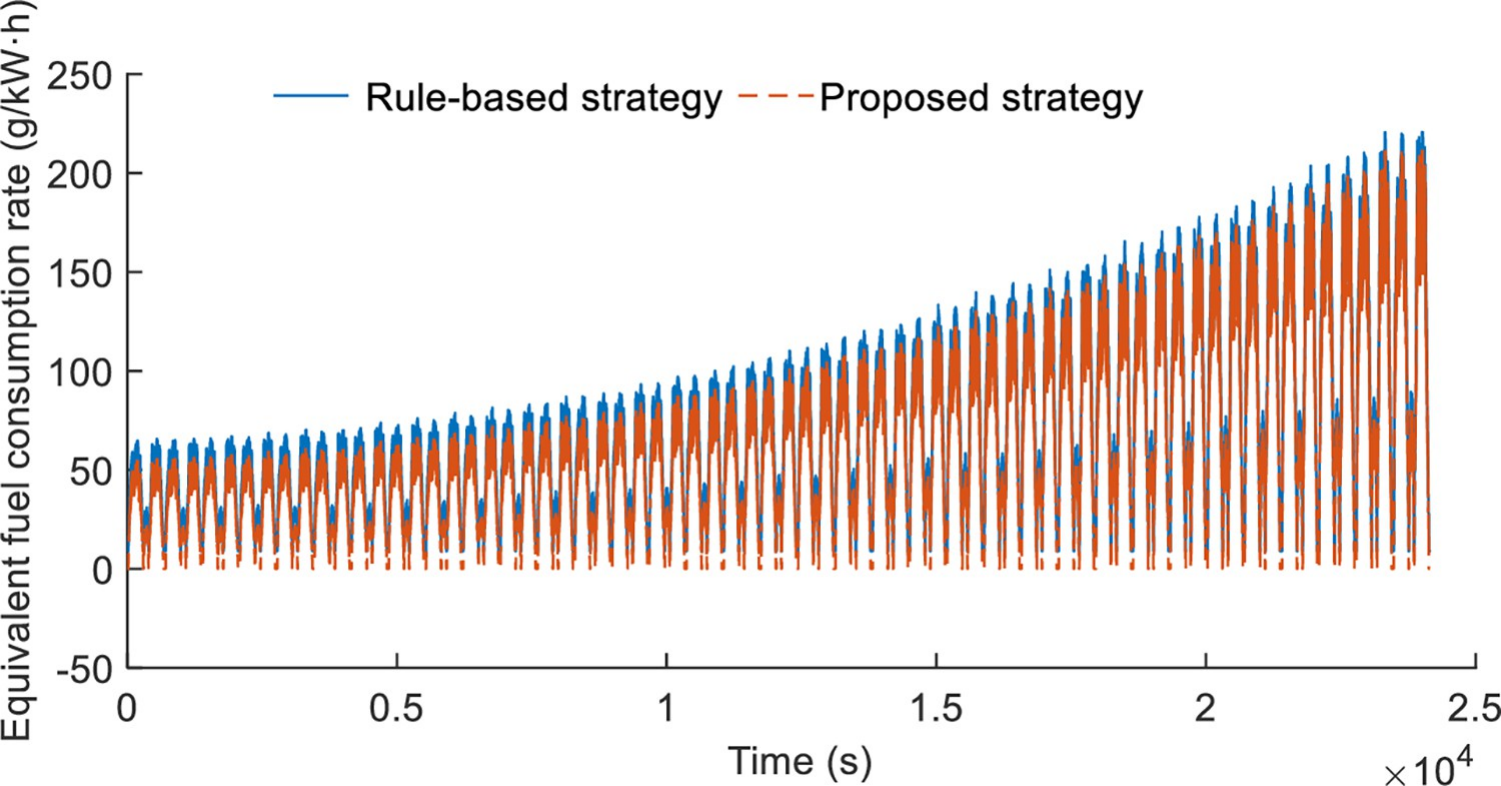
Comparison between rule-based and proposed strategy with equivalent fuel consumption rate.

**Fig 9:**
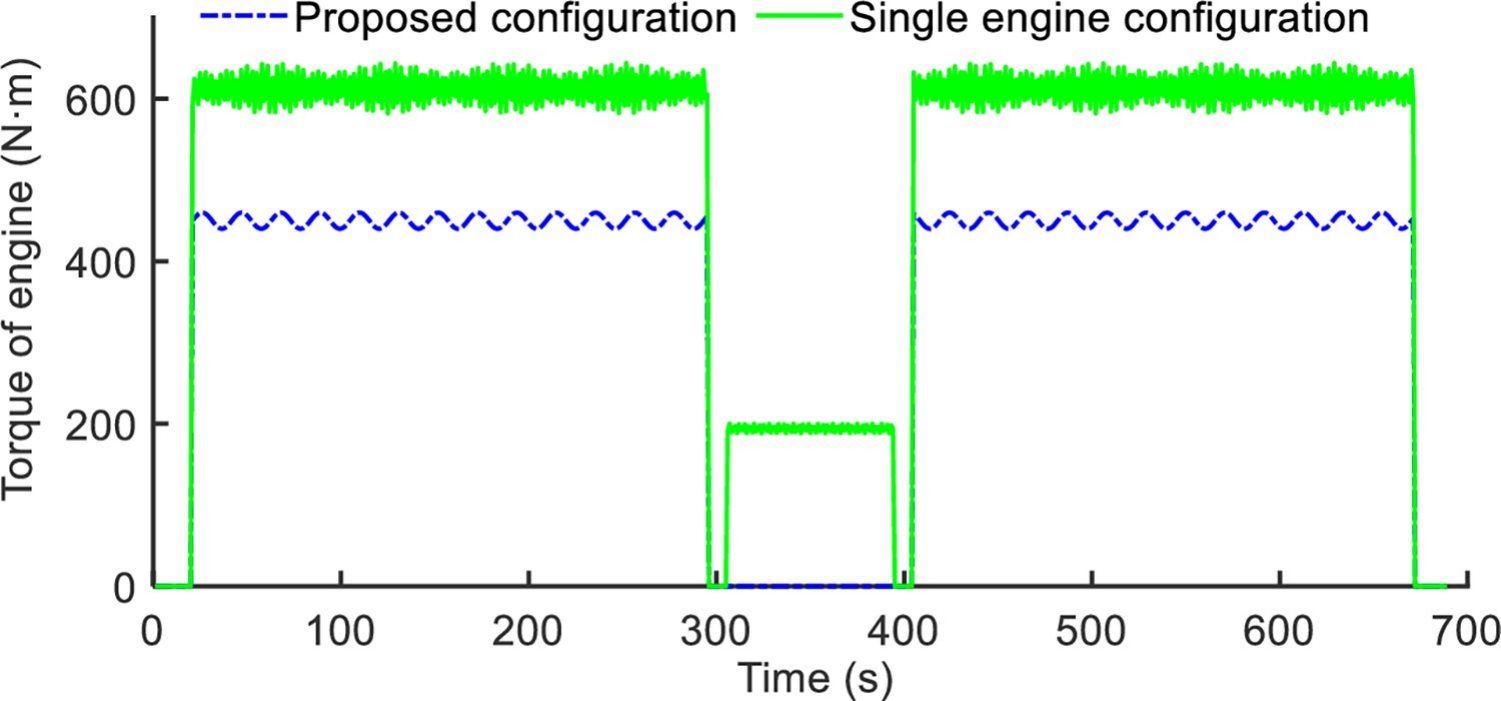
Comparison of engine torque fluctuations between proposed architecture and single engine.

This section discuss the adaptive equivalent-consumption minimization strategy’s fuel efficiency. A-ECMS is compared to the conventional rule-based (RB) system in order to show its effectiveness. Fig 8 displays the findings about the equivalent fuel consumption rate. The optimized torque distribution map is integrated into VCU through a look-up table, then the A-ECMS could be conducted in real-time along the accelerator pedal opening. The real-time implementation of the EMS, which contributes to the online application of the torque distribution in DPCTs, is greatly impacted by the reduction in computational complexity. Additionally, Table 4 lists the two strategies’ fuel efficiency. The suggested algorithm performs more effectively. For the typical rotary tillage working conditions, the average equivalent fuel consumption is reduced from 4.4919 to 4.1935. Meanwhile, the plowing working conditions reduction of equivalent fuel by 6.2%. Specifically, the proposed architecture of the distributed power unit has reduced torque fluctuations compared with a single engine in a tractor. The comparison of engine torque fluctuations between proposed configuration and traditional power system which is single engine mode is shown in Fig 9. The engine torque of proposed configuration could reduce torque ripple by 34.1%, that could extend the service life of the engine. In summary, the proposed EMS shows great strength of energy-saving and the balance of torque fluctuations with a single engine in a tractor. Besides, the power of engine and electric is more flexible to meeting the demand for tractor load, and to maintaining the engine efficiency, thereby improving fuel economy of the DPCT.

**Table 4:** Comparison of the fuel economy.

Test case	Equivalent fuel consumption		Terminal SOC
Case 1	Rule-based	4.7272	0.28
	A-ECMS	4.4523	0.28
	Variation(%)	6.2%	-
Case 2	Rule-based	4.4919	0.3
	A-ECMS	4.1935	0.3
	Variation(%)	7.1%	-

### Case 3: Simulation and test analysis

**Fig 10:**
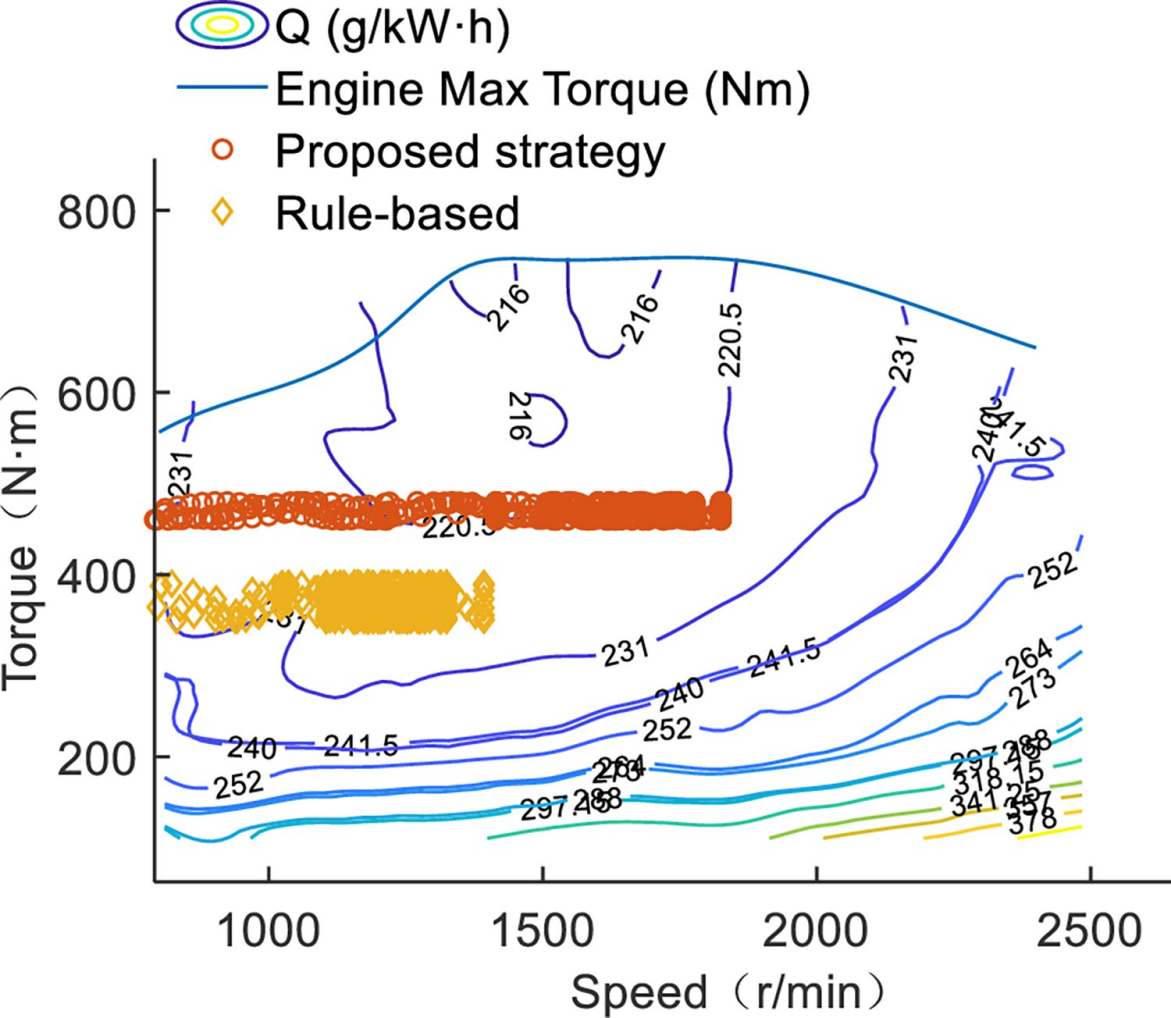
Comparison of engine operating points.

**Fig 11:**
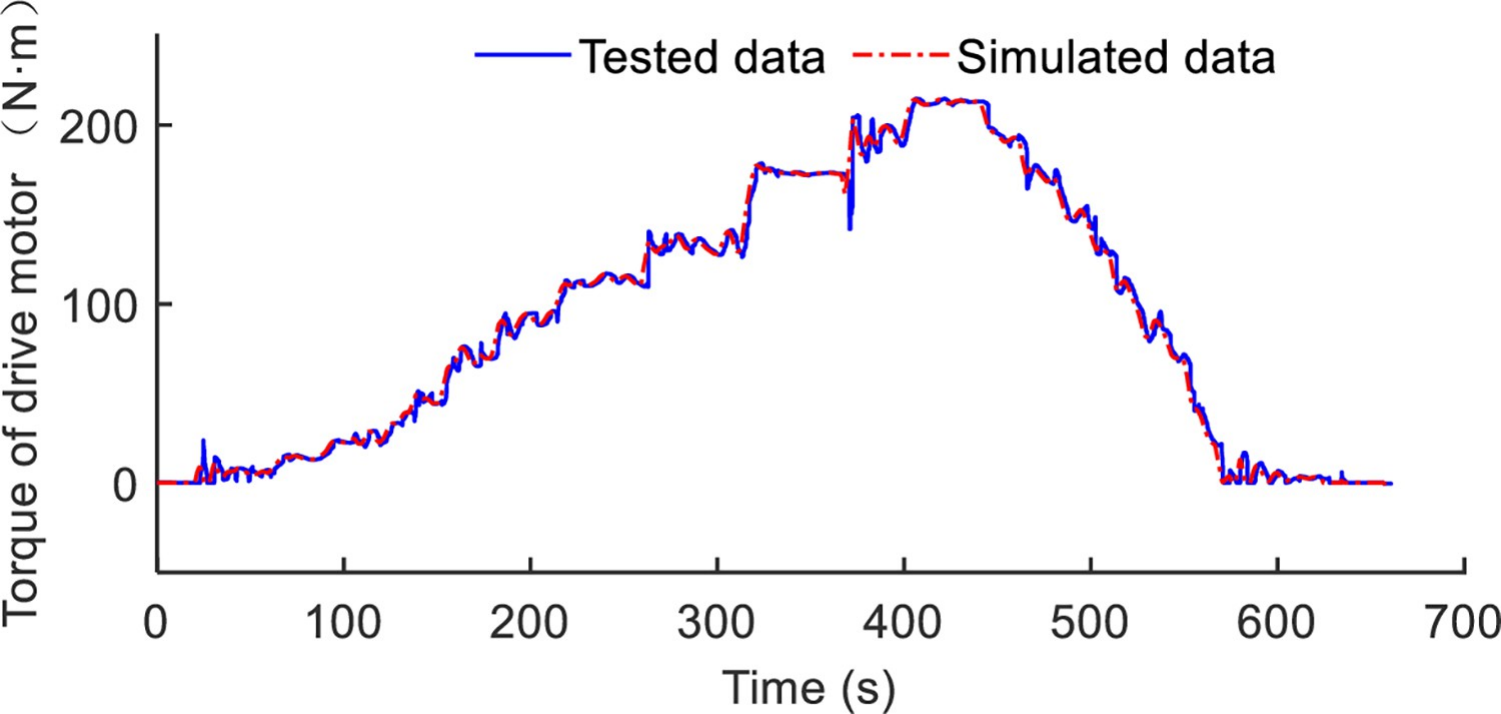
Comparison of drive motor torque between the experimental and simulation.

To embody the engine efficiency more intuitively, the engine working points are shown in Fig 10. The torque and speed corresponding to the fuel consumption of the engine between proposed strategy and rule-based are compared in tractors plowing conditions. In the proposed strategy, the engine is working in closer to the efficient range, and this is adjusted dynamically by ISG motor. The excess torque is used to generate electricity by ISG motor. But in rule-based strategy, the torque of engine is allocated based on power demand, which the efficiency of fuel efficiency could be improved further. As for experimental and simulation results comparison, Fig 11 is shown the troque distribution of drive motor in a drive cycle. The tested data is recorded by industrial computer when the drive motor is working in plowing operation test. At the same time, the load resistance and accelerator pedal opening signal are similarly added to the simulation model. The maximum relative error is 10.2%, in the operation of agriculture of tractor, the relative error is acceptable. The relative error may be occur in the following points. Firstly, the accuracy of industrial computer recorded should be further improved. Secondly, the simulation model should be more accurate, considering the delay of power system and friction loss. Lastly, the accelerator pedal opening signal between simulation and the actual applied is an error source. So the relative error between tested and simulated is acceptable in tractors plowing situation.

## Conclusions

This study investigated an A-ECMS adaptive power distribution approach to increase the fuel efficiency of the new architectural tractor. Firstly, the various power flow and electric machine modes were examined. The tractor optimization algorithm was built using a nonlinear load mode based on tractor dynamics. Additionally, the A-ECMS algorithm, in which the equivalency factor was nonlinearly improved, was used to increase fuel efficiency. Finally, the test bench platform was utilized to confirm the viability of the suggested technique. The applicability of results could be used in high-power tractors, they may replace the complicated gearbox to realize the technology of power-output device shift. And the A-ECMS could distribute the power efficiently. The outcomes demonstrated that:

1) The proposed tractor’s architecture could independently distribute the energy between the PTO and rear drive system verified by bench test. The DPCT was predisposed to combination power between engine and motor for power demand. It also tended to operate flexibly, allowing the ISG motor, drive motor, and engine to torque couple or decouple, as appropriate. When compared to a system with a single engine, the proposed architecture’s torque fluctuation was less, and balanced by motor.

2) When compared to the rule-based approach, the A-ECMS algorithm could lower the equivalent fuel consumption for conventional plowing by 6.2%. Additionally, the suggested technique exhibited a significant benefit in terms of energy saving in plowing operating situations. The online control law map’s streamlined computation indicated that it is appropriate for DPCTs’ online energy management.

## Abbreviations

A-ECMS: Adaptive-equivalent-consumption minimization strategy
DPCT: Dual-power coupling tractor
F_a_: Wind resistance
F_g_: Climbing resistance
F_A_: Acceleration resistance
F_r_: Rolling resistance
HDM: Hybrid drive mode
HPM: Hybrid PTO mode
ISG: Integrated Starter and Generator
*i*_o_: The reduction ratio of the final drive
*i*_1_: Reduction ratio in low gear
*i*_3_: The reduction ratio of the PTO gearbox
*J*_*i*_, *J*_*d*_: Inertia coefficient of the drive motor and ISG motor
J_e_: Inertia coefficient of engine
M: Mass of the total vehicle
PTO: Power take-off
P_dem_: Tractor demand power
P_bat_: The sum of battery output and input power
P_T_: Traction power of the tractor
R_int_: The internal resistance of the battery
r_r_: The equivalent radius of the tires
SM1/2: Single-drive motor mode
SOC: State of charge
T_out_: Total torque of the output
T_pto_: The output torque of PTO shaft
T_e_: Torque of engine
T_i_: Torque of ISG motor
V_x_: The longitudinal velocity of DPCT
V_oc_: Bus voltage of the battery
ω_w_: Angular velocity of wheels
ω_d_, ω_i_: Angular velocity of the drive motor and ISG motor
ω_e_: Angular velocity of the engine
$\dot{\omega_{e}}$: Angular acceleration of the engine
$\dot{\omega_{i}},\;\dot{\omega_{d}}$: Angular acceleration of ISG motor and drive motor
$\dot{\omega_{e}}$: Angular acceleration of the engine
θ: Slope gradient of the land
*η*_*d*_, *η*_*i*_: The efficiency of the Drive Motor and ISG Motor
